# Parental Opinions and Attitudes about Children’s Vaccination Safety in Silesian Voivodeship, Poland

**DOI:** 10.3390/ijerph15040756

**Published:** 2018-04-15

**Authors:** Bogumiła Braczkowska, Małgorzata Kowalska, Kamil Barański, Maksymilian Gajda, Tomasz Kurowski, Jan E. Zejda

**Affiliations:** 1 Department of Epidemiology, School of Medicine in Katowice, Medical University of Silesia, 40-055 Katowice, Poland; bbraczkowska@sum.edu.pl (B.B.); mkowalska@sum.edu.pl (M.K.); kbaranski@sum.edu.pl (K.B.); jzejda@sum.edu.pl (J.E.Z.); 2 Student Scientific Society Unit at the Department of Epidemiology, School of Medicine in Katowice, Medical University of Silesia, 40-055 Katowice, Poland; epikat@sum.edu.pl

**Keywords:** vaccination safety, adverse vaccine reaction, parental opinions

## Abstract

Despite mandatory vaccinations in Poland, the final decision on vaccination in children is taken by their parents or legal guardians. Understanding parents’ attitudes and opinions regarding vaccinations is essential for planning and undertaking extensive and properly targeted educational actions aimed at preventing their hesitancy. In 2016, a cross-sectional study was conducted in the Silesian Voivodeship (Poland) in 11 randomly selected educational institutions. The authors’ self-administered questionnaire contained 24 mixed-type questions. It was distributed among 3000 parents or legal guardians of children aged 6–13 years; prior consent of the relevant bioethics committee had been obtained. The response rate was 41.3% (*N* = 1239). Data were analysed using descriptive and analytical statistics, and focused on parental opinions regarding the safety of vaccines. Results of simple and multivariable analyses showed that perceived risk of adverse vaccine reaction (AVR), contraindications and perception of the qualification procedure for vaccination as substandard were significant factors associated with the rating of children’s vaccination as unsafe (*p* < 0.001). Respondents with a lower level of education, compared with those with higher, more often declared vaccinations to be safe (*p* = 0.03); however, results of multivariable analysis did not confirm that effect. AVR occurrence, finding of contraindication to vaccinations and perception of qualification procedure for vaccination were found to be the most important factors responsible for influencing general public opinions in the field of vaccination safety.

## 1. Introduction

Although the epidemiological situation of infectious diseases in Poland is classified as favourable and relatively stable, it requires constant surveillance and monitoring of trends [1]. Epidemiological surveillance data indicate a marked increase in the incidence of pertussis and measles, reaching over 100 new cases of either disease per year. The interpretation of that finding links the spread of both diseases with low vaccination rates in specific subpopulations [1]. In Poland, both diseases are covered by a mandatory vaccination program and in spite of the average vaccination rate of 97.6% of children [2], experts believe that improved surveillance and appropriate preventive actions are needed [3]. It is also believed that the level of the population’s acceptability of vaccination is crucial to the achievement of high vaccination rates [4]. In spite of regulatory enforcement regarding vaccinations in Poland, as well as in other European Union countries, the final decision on vaccination in children is taken by their parents or legal guardians. Among the important determinants of this decision are factors such as: personality, previous life experiences, perceptions of vaccination in terms of “standard”, customs or natural behaviours. Other pertinent factors include education and knowledge about the benefits and contraindications. Finally, their decision may result in carrying out, delay or even refusal of the vaccination [5]. Understanding parents’ attitudes and opinions regarding vaccinations is essential for planning and undertaking extensive and properly targeted educational actions aimed at preventing their hesitancy. The aim of this paper was to identify the parental opinions and their determinants on the safety of children vaccination in Poland. The choice of research region (Silesian Voivodeship) was dictated by one of the highest rates of childhood vaccine hesitancy in Poland [6]. To our knowledge, the present study is the first conducted in the Silesian Voivodeship and included the largest sample size of Polish population in this research area.

## 2. Materials and Methods

A population-based questionnaire survey was conducted in 2016, in one of the largest regions of Poland, the Silesian Voivodeship, with nearly 10% of the population eligible for vaccination. Both the number of children and the percentage of vaccinations in the studied region are representative for Poland [2,7]. Three thousand parents or legal guardians of children aged 6–13 years attending 11 (out of 323) randomly selected (cluster randomisation) schools and kindergartens in Katowice, Zabrze and Ruda Śląska were invited to participate in this study. They were asked to fill out (anonymously and voluntarily) our questionnaire. At the beginning of the questionnaire, preliminary information was provided on the anonymity, purpose of the study and completion time. The main part of the questionnaire included 24 mixed-type questions concerning, among others, the safety of vaccinations and combined vaccines, current vaccination regimens, adverse vaccine reaction (AVR), presence of contraindications to vaccination, evaluation of qualification procedure, preferred information sources, and the role of health workers in providing information on vaccination and qualification of children to immunization. We did not include any questions collecting identifiable data. Bearing in mind the widespread decline of interest in participation in survey-based studies, in order to maximise the response rate, every invited person was told about the purpose of this study. We designed a friendly layout and used simple language. In this paper, we focus on questions regarding the safety of used vaccines, especially question number 10 simplified into two categories: “safe” versus “not convinced” merged with “dangerous” The questionnaire was validated by testing a group of 35 parents two times within a period of three weeks. The repeatability of answer to particular questions was evaluated using Kappa Cohen statistics [8,9]. A very good repeatability of answers to all selected questions is shown in Table 1. The study protocol was approved by the Bioethical Committee of the Medical University of Silesia in Katowice, Poland (KNW/0022/KB/246/15). The English, not validated, version of the questionnaire is available upon reasonable request from the corresponding author.

Taking into an account the non-normal distribution of age in the study group, we used the Mann–Whitney U-test to assess differences in responses to questions regarding vaccination safety. A chi-square test was used for the assessment of diversity of opinion on the safety of vaccination in groups: younger vs. older subjects (median value criterion), lower vs. higher education level, smaller vs. larger place of residence, worse vs. better the economic status of the family, declared ever AVR (yes vs. no), contraindications to vaccination (yes vs. no), evaluation of the qualification for vaccination (question number 19, categorised as good or bad). Finally, the results of simple analyses were verified by two models of binomial logistic regression predicting “declared safety of vaccination” (question number 10 recoded as binomial variable, as described previously). Stepwise regression based on Akaike’s Information Criterion was used to choose the best model. To assess regression model fit, we chose McFadden’s pseudo R-squared measure (it was calculated with pR2 function from “pscl” package of R software). For all analyses, procedures available in R 3.2.5 software (R Core Team, Vienna, Austria) were used [10]. Missing data were excluded from all analyses and the level of significance was set at *p* < 0.05 a priori. 

## 3. Results

The questionnaire was filled out by 1239 parents or legal guardians of pre-school or school children (response rate of 41.3%). The age of the subjects ranged from 22 to 80 years, with median value of 37 and interquartile range (IQR) of 7 years. The median age of the first child was 10 years, of the second one it was 7 years, and of the third child it was 6 years. Table 2 contains other descriptive statistics identifying the examined group. Two thirds of subjects (64.3%) were of the opinion that children’s vaccinations are safe, 32.3% of respondents declared AVR’s occurrence mostly reported as: redness, pain and swelling at the injection site (27%), fever (24.9%) and anxiety, continuous crying or screaming (9.0%), somnolence (8.7%) and lack of appetite (8.2%).

**Figure 1:**
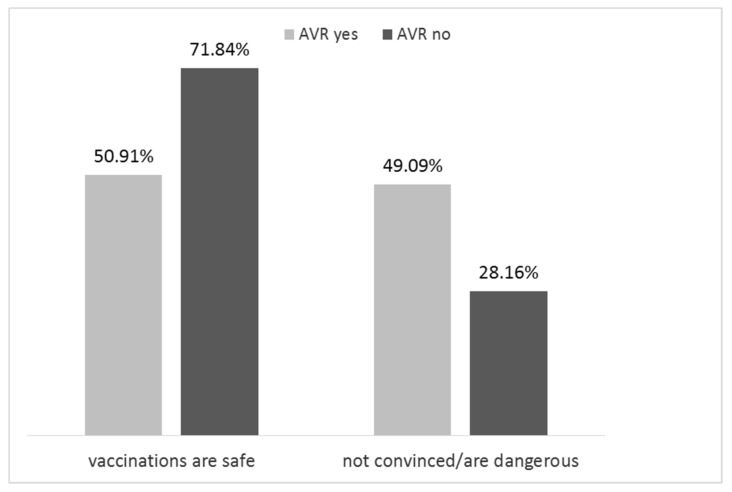
Percentage of parents’ responses to a questionnaire regarding the safety of vaccination in relation to ever occurred adverse vaccine reaction (AVR) in any child.

According to the results of simple analysis, the level of confidence in vaccination safety was statistically significant associated with the negative evaluation of qualification procedure for vaccination, occurrence of AVR, presence of contraindications to immunization. Better-educated people less frequently perceived vaccination as safe, compared to those with a lower level of education (*p* = 0.03). Only parents who reported AVR (4 children) indicated that vaccinations might be dangerous (Figure 1). Other explanatory variables were not relevant to the question of the safety of children’s vaccinations. 

The results of the first model of multivariable analysis (Table 3, part A) suggest that the most important determinants of negative parental opinion about vaccination’s safety were: the occurrence of AVR in any of the vaccinated children, presence of contraindications as well as negative evaluation of qualification procedures.

Supplementary Materials Tables S1 and S2 provide results of simple analyses showing the diversity of specific answers according to education, economic status of the families, number of children, and the occurrence of any AVR in the child.

Table 3 (part B) shows the results of the second multivariable analysis addressing the association of parental opinions with considering vaccines as safe. Those who did not share the opinion about the safety of vaccination were unlikely to consider them as an important method for the prevention of infectious diseases and to perceive the current immunization strategy as reasonable. They more often denied that vaccinations provide long-term immunity and declared that the evidence of their efficacy is insufficient. Opponents of vaccination safety more often stated that vaccination should not be performed too early and that their number was too high and should be reduced. They were less likely to be of the opinion that information on AVR is sufficient. The negative attitude of this group of respondents towards the topic of combined vaccines were also highlighted, their rarely agreeing statement about their safety and lower price than multiple vaccinations.

## 4. Discussion

Safety of immunization remains one of the priorities of the World Health Organization (WHO). In 1999, WHO appointed a special agenda called “The Global Advisory Committee on Vaccine Safety” (GACVS). GACVS provides independent, authoritative, scientific advice on vaccine safety issues of global or regional concern in case of the WHO recommended immunization [11]. According to the current “2017 Assessment Report of the Global Vaccine Action Plan Strategic Advisory Group of Experts on Immunization”, progress in the field is not satisfactory [12].

A review of available literature indicated that parents’ beliefs about the possibility of serious, distant AVRs belong to important factors strongly associated with vaccination hesitancy. The concerns of caregivers most often focus on the composition of vaccines as well as side effects, being usually related to their negative experiences with vaccination [6,13,14].

Our study focused on the opinions of parents or legal guardians of children regarding the safety of vaccines used in Poland. This is one of the important determinants of parents’ attitudes towards immunizations, including approaches that are responsible for evading compulsory or recommended vaccinations. The public opinion survey method used in this paper is considered to be a pertinent tool for routine surveillance addressing preventive measures including active immunization methods of the population [15]. 

The majority of the subjects in our study (62%) believed that vaccines currently used in children are fully safe. However, this figure is lower than that obtained in a study conducted in Poland by the “Public Opinion Research Center” (CBOS) on a randomly selected, representative group of adults (*N* = 977) in 2017, where 73% of the respondents confirmed the safety of vaccination of children compared to 74% in 2013 [16]. Comparable results were obtained in studies on parents’ attitudes towards children’s vaccination aged up to 3 years old in selected European countries [15]. It has been shown that, similarly as in Poland, in Norway, Spain and Sweden, the attitudes of parents to vaccinations are generally positive, although the results vary somewhat depending on the involvement of governmental institutions in the implementation of vaccination, countries’ demographic situation as well as participants’ socio-economic status and the method of recruitment [15].

The important findings of our study are that every third respondent (about 34%) did not believe in the safety of vaccination in children and it cannot be ruled out that most of them may refuse to comply with preventive vaccines regimens in Poland. These results are similar to those reported by CBOS in August 2017, where 37% of participants revealed concerns about possibility of vaccinations’ serious side effects [16]. Another Polish study showed that 20% of parents were concerned about the safety of vaccines [15]. From that point of view, our findings do not reveal a promising trend. In Poland, the situation is not homogenous across the population. In 2015, one of the highest rates of vaccination hesitancy was registered in the Silesian Voivodeship, 4.69/1000 compared with the average country level of 2.58/1000 people 0–19 years old [6]. Increased rates may partially result from the growing activity of anti-vaccination movements and their frequent campaigns denoting vaccines as harmful [1]. Such possibility seems to be supported by data published by Polish National Sanitary Inspection, showing that in relation to every third unvaccinated child (32%) the parents’ decision was due to the influence of anti-vaccination movements [17]. In this context, a particularly disturbing phenomenon in Poland is the overt criticism of vaccination and their appropriateness by the medical professionals and decision makers. Their voice can be heard in the media (including nationwide television) and even in parliament [18]. Some recent reports on approach to vaccination in Poland show a need to promote evidence-based knowledge on vaccinations among physicians and nurses [19,20]. According to our analysis, parents’ opinions regarding the vaccination’s safety significantly depended on whether the doctor had ever found medical contraindications for vaccination.

We observed differences in the opinions of parents regarding the safety of children’s vaccinations related to the level of parent’s education, past vaccine-related experiences and opinions. Parents with a higher level of education were more suspicious of vaccination; however, it was not confirmed in the multivariable analysis. Nevertheless, similar results have been reported by other authors [21,22].

On the other hand, our results demonstrated that factors such as place of residence, age of the respondents, the economic status of families and the number of children in the family had no significant effect on the declared opinions about safety of vaccination. Similarly, the report published in 2017 by CBOS revealed that the impact of socio-demographic variables on opinion diversification was small [16]. According to our results, negative results of qualification process for vaccination as well as physician diagnosis of contraindications could be very important factors of perceiving vaccinations’ safety. Moreover, it appears that another important factor related to perception of immunization safety was the experience of AVR in any of their own children. This is in line with the published evidence [23,24].

Insufficient contact with the physicians, especially the lack of reliable information provided by specialists regarding the safety, mechanism of action and the effectiveness of the vaccinations are among important factors that influence parents’ attitude to vaccination of children [25]. The situation also seems to be aggravated by the poor rating of the qualification system. According to our results, nearly 30% of respondents rated this process negatively. 

Misconceptions and lack of knowledge, including the etiology of infectious diseases and vaccination, have contributed to the development of so-called anti-vaccination movements [4,6,13,19]. It seems that in order to reduce the proportion of people who avoid vaccination, it is necessary to improve the relationship between the physician and the parent in terms of communicating the risks and safety of vaccination [4]. There is an important argument for physicians who take preventive care for children, also in terms of health education, organization and legislation. 

Our study has some obvious limitations. The most important one is related to the response rate. It cannot be ruled out that non-responder bias occurred. However, such a doubt is pertinent in other population-based surveys on the subject and a large sample of our study seems to add some credibility to our findings. They point to a need for further health education efforts at least in the population under study, including both young families and health professionals. Another potential limitation stems from the method used to assess opinions and perceptions. This is a general concern of many questionnaire studies. However, a good repeatability of answers to our questionnaire supports a validity of the results. It is also of interest that our findings correspond with the results of other studies addressing parental opinions and attitudes about children’s vaccination safety. 

## 5. Conclusions

The majority of surveyed Polish parents perceived vaccinations as safe. The most important factors responsible for parental opinions of vaccination safety in children appear to be: AVR occurrence, finding of contraindication to vaccinations and perception of adopted method of children qualification to vaccination. For the improvement of the epidemiological situation of vaccination, i.e., to reduce the percentage of unvaccinated children, it is necessary to continue educating parents in this field with the active participation of medical doctors.

## Figures and Tables

**Table 1:** Repeatability of answers to selected questions, values of Kappa-Cohen statistics along with 95% confidence intervals (95% CI).

Question	Κ Cohen Statistic	95% CI
10a. Vaccinations are completely safe	1.0	1.0–1.0
10b. I’m not convinced about their safety		
10c. They are dangerous		
11a. Vaccinations are a very important method for the prevention of infectious diseases	0.78	0.39–1.0
11b. The evidence of vaccinations efficacy is insufficient	1.0	1.0–1.0
11c. Vaccinations did not provide long-term immunity	1.0	1.0–1.0
11d. The fact of being sick with an infectious disease results in a better immunity than vaccination	1.0	1.0–1.0
11e. The realization of vaccination is indicative of parents’ concern for children’s health	0.92	0.79–1.0
11f. The current vaccination strategy is reasonable	1.0	1.0–1.0
11g. Vaccination should not be performed too early (e.g., just after birth)	0.91	0.72–1.0
11h. The number of vaccinations is too high and should be reduced	0.94	0.85–1.0
11i. The vaccination costs outweigh the benefits	1.0	1.0–1.0
11j. Education in this subject is sufficient	1.0	1.0–1.0
11k. Information on the unwanted post-vaccination reactions is sufficient	1.0	1.0–1.0
12a. Currently used combined vaccines are safe	1.0	1.0–1.0
12b. Currently used combined vaccines are as effective as single vaccines	1.0	1.0–1.0
12c. Currently used combined vaccines are cheaper than multiple vaccinations	1.0	1.0–1.0
12d. Currently used combined vaccines cause less stress and injection-related pain in children	1.0	1.0–1.0
12e. Currently used combined vaccines are dangerous as vaccinations are associated with severe illness	1.0	1.0–1.0

**Table 2:** Opinion about safety of children’s vaccinations according to selected variables, with statistical significance of differences (only complete answers).

Variables		Answers Regarding Vaccination Safety			
		All Complete	Safe	Not Safe	*p*
		*N* = 1187	*N* = 763 (64.3%)	*N* = 424 (35.7%)	
Person filling out the questionnaire	mother	87.4%	64.8%	35.2%	0.1
	father	10.4%	56.9%	43.1%	
	other (legal guardian)	1.3%	80.0%	20.0%	
	missing data	0.9%	72.7%	27.3%	
Level of parent’s education *	lower	47.0%	67.4%	32.6%	0.03
	higher	52.0%	60.9%	39.1%	
	missing data	1.0%	91.7%	8.3%	
Respondent’s age	median (IQR)	37 (8)	37 (8)	37 (6)	0.8
	missing data	4.0%	3.8%	4.2%	
Place of child’s residence	small city and village	13.9%	68.5%	31.5%	0.2
	big city	83.4%	63.2%	36.8%	
	missing data	2.7%	75.0%	25.0%	
Economic status of the family	worse (bad and quite good)	24.5%	62.2%	37.8%	0.5
	better (good and very good)	73.2%	64.8%	35.2%	
	missing data	2.3%	70.4%	29.6%	
Number of children in the family	one child	31.2%	63.5%	36.5%	0.8
	more	67.8%	64.6%	35.4%	
	missing data	1.0%	66.7%	33.3%	
Adverse vaccine reaction (AVR) occurrence	ever	32.3%	50.9%	49.1%	<0.001
	never	60.7%	71.8%	28.2%	
	not remember	5.6%	56.7%	43.3%	
	missing data	1.3%	75.0%	25.0%	
Contraindication	presence	10.3%	45.1%	54.9%	<0.001
	absence	89.5%	66.7%	33.3%	
	missing data	0.3%	0.0%	100.0%	
Evaluation of qualification to vaccinations (simplified)	good	68.2%	74.4%	25.6%	<0.001
	bad	29.1%	40.6%	59.4%	
	missing data	2.7%	62.5%	37.5%	

Legend: *p*—level of significance in tests (respectively chi-square for qualitative or Mann–Whitney U for quantitative variables); *—lower: primary, vocational or secondary and higher: university degree; IQR—interquartile range.

**Table 3:** Results of multivariable logistic regression (two models: part A and B, respectively) to determine factors associated with parental negative opinion regarding the safety of vaccination in children. Dependent variable: question 10 (safety of vaccinations in children) coded as: No vs. Yes.

**Part A.**			
**Independent variables (*with coding*)**	OR	95% CI	*p*
AVR occurrence *yes vs. no*	2.01	1.5–2.7	<0.001
Contraindication to vaccination *presence vs. absence*	2.13	1.4–3.3	0.001
Evaluation of qualification to vaccinations *good vs. bad*	3.86	2.9–5.2	<0.001
Place of child’s residence *small city and village vs. big city*	0.76	0.5–1.1	0.2
Respondent’s age (*in years*)	1.01	0.9–1.0	0.3
Respondent’s level of education *ordinal, from lower to higher*	0.92	0.7–1.1	0.5
Number of children in the family (continues variable)	0.98	0.8–1.2	0.8
Economic status of the family *ordinal, from higher to lower*	0.88	0.7–1.1	0.3
207 observations deleted due to missing data. McFadden’s pseudo R^2^ = 0.21. AIC = 1230.5			
**Part B.**			
**Independent variables (answers to questions No. 11 and 12); coded: *Yes vs. No***	OR	95% CI	*p*
Vaccinations are a very important method for the prevention of infectious diseases	0.15	0.04–0.4	0.002
The evidence of vaccinations efficacy is insufficient	3.60	2.3–5.6	<0.001
Vaccinations did not provide long-term immunity	1.51	1.03–2.2	0.03
The fact of being sick with an infectious disease results in a better immunity than vaccination	1.23	0.8–1.9	0.4
The realization of vaccination is indicative of parents’ concern for children’s health	0.88	0.4–1.8	0.7
The current vaccination strategy is reasonable	0.51	0.3–0.7	0.001
Vaccination should not be performed too early (e.g., just after birth)	1.85	1.2–2.9	0.006
The number of vaccinations is too high and should be reduced	2.34	1.4–3.8	0.001
The vaccination costs outweigh the benefits	0.87	0.5–1.4	0.6
Education in this subject is sufficient	1.22	0.8–2.0	0.4
Information on the AVRs is sufficient	0.29	0.2–0.5	<0.001
Currently used combined vaccines are safe—lower risk of AVRs	0.35	0.2–0.5	<0.001
Currently used combined vaccines are as effective as single vaccines	0.87	0.6–1.3	0.5
Currently used combined vaccines are cheaper than multiple vaccinations	0.51	0.3–0.8	0.001
Currently used combined vaccines cause less stress and injection-related pain in children	3.0	1.8–5.2	<0.001
Currently used combined vaccines are dangerous as vaccinations are associated with severe illness	1.15	0.7–1.9	0.6

298 observations deleted due to missing data. McFadden’s pseudo R^2^ = 0.43. AIC = 908.66. Legend: OR—odds ratio; CI—confidence interval; *p*—level of significance; AIC—Akaike information criterion.

## Supplementary Materials

The following are available online at http://www.mdpi.com/1660-4601/15/4/756/s1, Table S1: Distribution of parental opinions on vaccinations in children according to occurrence of AVR, education, economic status, family size and age of respondents (along with significance of chi^2^ test), Table S2: Distribution of parental opinions on combined vaccines in children according to occurrence of AVR, education, economic status, family size and age of respondents (along with significance of chi-square test).
