# Supplementary material for: Parental Opinions and Attitudes about Children’s Vaccination Safety in Silesian Voivodeship, Poland
Source: Int J Environ Res Public Health. 2018 Apr 15;15(4):756. doi: 10.3390/ijerph15040756 (PMC5923798; doi:10.3390/ijerph15040756)
Supplement: Supplementary file 1 [file ijerph-15-00756-s001.zip › Table S1.docx]

**Appendix A.** Distribution of parental opinions on vaccinations in children according to occurrence of AVR, education, economic status, family size and age of respondents (along with significance of chi^2^ test).

| Positive answers to question 11 | AVR occurrence ever (%) | | p | Level of education of respondent (%) | | p | Economic status of the family (%) | | p | Number of children in the family (%) | | p | Age of the respondent (%) | | p |
| --- | --- | --- | --- | --- | --- | --- | --- | --- | --- | --- | --- | --- | --- | --- | --- |
|  | yes | no |  | lower | higher |  | worse | better |  | > 1 | 1 |  | older | younger |  |
| Vaccinations are a very important method for the prevention of infectious diseases | 92.78 | 95.73 | 0.1 | 93.39 | 94.17 | 0.8 | 94.18 | 93.0 | 0.3 | 93.71 | 93.72 | 0.9 | 93.61 | 94.06 | 0.8 |
| The evidence of vaccinations efficacy is insufficient | 24.53 | 18.5 | 0.01 | 24.72 | 18.14 | 0.001 | 20.92 | 20.91 | 0.9 | 20.85 | 21.49 | 0.4 | 19.96 | 21.52 | 0.1 |
| Vaccinations did not provide long-term immunity | 32.16 | 25.59 | 0.002 | 27.22 | 27.24 | 0.8 | 26.32 | 28.62 | 0.3 | 27.12 | 27.82 | 0.2 | 26.77 | 27.67 | 0.6 |
| The fact of being sick with an infectious disease results in a better immunity than vaccination | 17.55 | 14.94 | 0.002 | 17.65 | 14.4 | 0.3 | 16.24 | 13.59 | 0.1 | 15.32 | 16.62 | 0.2 | 14.29 | 16.89 | 0.2 |
| The realization of vaccination is a indicative of parents' concern for children's health | 87.99 | 92.19 | 0.05 | 88.59 | 91.28 | 0.04 | 90.4 | 89.12 | 0.7 | 90.26 | 89.52 | 0.6 | 90.11 | 89.87 | 0.9 |
| The current vaccination strategy is reasonable | 59.15 | 78.44 | <0.001 | 73.66 | 68.97 | 0.1 | 72.25 | 69.10 | 0.5 | 71.63 | 71.04 | 0.9 | 69.75 | 72.68 | 0.4 |
| Vaccination should not be performed too early  (eg just after birth) | 26.93 | 14.72 | <0.001 | 19.27 | 18.81 | 0.6 | 19.01 | 18.25 | 0.4 | 19.25 | 19.07 | 0.5 | 18.76 | 19.30 | 0.01 |
| The number of vaccinations is too high and should be reduced | 26.53 | 13.91 | <0.001 | 19.15 | 16.85 | 0.5 | 16.69 | 21.18 | 0.1 | 18.07 | 18.21 | 0.6 | 17.39 | 19.04 | 0.1 |
| The vaccination costs outweigh the benefits | 17.46 | 14.5 | 0.1 | 20.89 | 12.04 | <0.001 | 14.68 | 18.95 | <0.001 | 16.65 | 15.11 | 0.04 | 11.95 | 19.01 | <0.001 |
| Education in this subject is sufficient | 19.10 | 22.33 | 0.4 | 27.47 | 15.62 | <0.001 | 21,21 | 18,62 | 0,3 | 20.78 | 22.55 | 0.02 | 21.32 | 20.86 | 0.07 |
| Information on the AVR reactions is sufficient | 25.73 | 31.21 | 0.007 | 31.16 | 27.35 | <0.001 | 30,13 | 27,18 | 0,1 | 28.1 | 32.05 | 0.3 | 28.71 | 30.20 | 0.7 |
