# Supplementary material for: Parental Opinions and Attitudes about Children’s Vaccination Safety in Silesian Voivodeship, Poland
Source: Int J Environ Res Public Health. 2018 Apr 15;15(4):756. doi: 10.3390/ijerph15040756 (PMC5923798; doi:10.3390/ijerph15040756)
Supplement: Supplementary file 1 [file ijerph-15-00756-s001.zip › Table S2.docx]

**Appendix B.** Distribution of parental opinions on combined vaccines in children according to occurrence of AVR, education, economic status, family size and age of respondents (along with significance of chi^2^ test).

| **Positive answers to question 12** | AVR occurrence ever (%) | | p | Level of respondent’s education (%) | | p | Economic status of the family (%) | | p | Number of children in the family (%) | | p | Age of the respondent (%) | | p |
| --- | --- | --- | --- | --- | --- | --- | --- | --- | --- | --- | --- | --- | --- | --- | --- |
|  | yes | no |  | lower | higher |  | worse | better |  | > 1 | 1 |  | older | younger |  |
| Currently used combined vaccines are safe – lower risk of AVR | 43.42 | 57.20 | <0.001 | 53.23 | 50.48 | 0.6 | 54.64 | 44.18 | 0.007 | 51.23 | 53.37 | 0.08 | 50.66 | 52.79 | 0.6 |
| Currently used combined vaccines are as effective as single vaccines | 55.26 | 62.08 | 0.06 | 56.07 | 60.13 | 0.2 | 59.67 | 53.24 | 0.02 | 57.68 | 60.0 | 0.1 | 54.11 | 61.33 | 0.04 |
| Currently used combined vaccines are cheaper than multiple vaccinations | 27.0 | 36.83 | 0.002 | 40.54 | 28.52 | <0.001 | 34.65 | 33.80 | 0.1 | 34.53 | 33.61 | 0.5 | 37.11 | 31.58 | 0.05 |
| Currently used combined vaccines cause less stress and caused by injection pain in children | 89.21 | 87.86 | 0.7 | 82.48 | 91.55 | <0.001 | 88.59 | 83.56 | 0.05 | 88.37 | 85.83 | 0.4 | 89.41 | 86.54 | 0.03 |
| Currently used combined vaccines are dangerous as vaccinations are associated with severe illness | 19.61 | 11.99 | <0.001 | 17.95 | 11.42 | <0.001 | 14.62 | 13.04 | 0.03 | 14.73 | 13.70 | 0.8 | 14.54 | 14.06 | 0.3 |
